# Molecular Mechanisms of ZC3H12C/Reg-3 Biological Activity and Its Involvement in Psoriasis Pathology

**DOI:** 10.3390/ijms22147311

**Published:** 2021-07-07

**Authors:** Mateusz Wawro, Jakub Kochan, Weronika Sowinska, Aleksandra Solecka, Karolina Wawro, Agnieszka Morytko, Patrycja Kwiecinska, Beata Grygier, Mateusz Kwitniewski, Mingui Fu, Joanna Cichy, Aneta Kasza

**Affiliations:** 1 Department of Cell Biochemistry, Faculty of Biochemistry, Biophysics and Biotechnology, Jagiellonian University, 30-387 Krakow, Poland; mateusz.wawro@uj.edu.pl (M.W.); jakub.kochan@uj.edu.pl (J.K.); weronika.sowinska@doctoral.uj.edu.pl (W.S.); aleksandra.solecka@doctoral.uj.edu.pl (A.S.); karolina.wawro@gmail.com (K.W.); 2 Department of Immunology, Faculty of Biochemistry, Biophysics and Biotechnology, Jagiellonian University, 30-387 Krakow, Poland; auzarowska@wp.pl (A.M.); patrycja.kwiecinska@uj.edu.pl (P.K.); beata.grygier@uj.edu.pl (B.G.); mateusz.kwitniewski@uj.edu.pl (M.K.); joanna.cichy@uj.edu.pl (J.C.); 3 Department of Biomedical Science and Shock/Trauma Research Center, School of Medicine, University of Missouri-Kansas City, Kansas City, MO 64110, USA; fum@umkc.edu

**Keywords:** ZC3H12/MCPIP/Regnases, transcripts turnover, RNases, inflammation, psoriasis

## Abstract

The members of the ZC3H12/MCPIP/Regnase family of RNases have emerged as important regulators of inflammation. In contrast to Regnase-1, -2 and -4, a thorough characterization of Regnase-3 (Reg-3) has not yet been explored. Here we demonstrate that Reg-3 differs from other family members in terms of NYN/PIN domain features, cellular localization pattern and substrate specificity. Together with Reg-1, the most comprehensively characterized family member, Reg-3 shared IL-6, IER-3 and Reg-1 mRNAs, but not IL-1β mRNA, as substrates. In addition, Reg-3 was found to be the only family member which regulates transcript levels of TNF, a cytokine implicated in chronic inflammatory diseases including psoriasis. Previous meta-analysis of genome-wide association studies revealed Reg-3 to be among new psoriasis susceptibility loci. Here we demonstrate that Reg-3 transcript levels are increased in psoriasis patient skin tissue and in an experimental model of psoriasis, supporting the immunomodulatory role of Reg-3 in psoriasis, possibly through degradation of mRNA for TNF and other factors such as Reg-1. On the other hand, Reg-1 was found to destabilize Reg-3 transcripts, suggesting reciprocal regulation between Reg-3 and Reg-1 in the skin. We found that either Reg-1 or Reg-3 were expressed in human keratinocytes in vitro. However, in contrast to robustly upregulated Reg-1 mRNA levels, Reg-3 expression was not affected in the epidermis of psoriasis patients. Taken together, these data suggest that epidermal levels of Reg-3 are negatively regulated by Reg-1 in psoriasis, and that Reg-1 and Reg-3 are both involved in psoriasis pathophysiology through controlling, at least in part different transcripts.

## 1. Introduction

The ZC3H12/MCPIP/Regnase family of four members (ZC3H12A-D/MCPIP1-4/Regnase1-4) was discovered quite recently. First reports pointing out an anti-inflammatory role of ZC3H12A/MCPIP-1/Regnase-1 were published in 2009 [1,2]. Since then it was shown that Regnase-1 (Reg-1) is an RNase involved in the degradation of transcripts for proinflammatory factors, viral nucleic acids and precursor forms of microRNAs [3,4,5]. Few years later the RNase activities and the anti-inflammatory roles of ZC3H12D/MCPIP4/Reg-4 and ZC3H12B/MCPIP2/Reg-2 were reported. These proteins contain a CCCH type zinc finger responsible for their interaction with RNA. Their nucleolytic activity is based on the presence of a Mg2+-dependent catalytic NYN/PIN-like domain [6,7]. The amino acids sequence homologies of the NYN domains of Reg-2 and Reg-4 compared to Reg-1 are 83.2%, and 75.3%, respectively [5,8]. Reg-1, -2 and -4 interact with transcripts through the recognition of a hairpin structure present in their 3′UTRs [6,7]. The involvement of proteins from the ZC3H12 family in the regulation of inflammation occurs not only through their RNase activity. Reg-1 modulates also cell signaling by removing ubiquitin moieties from TRAF2, TRAF3, and TRAF6 [9]. Similarly, overexpression of Reg-4 significantly inhibits TLR2 and TLR4 activation-induced signaling, likely through deubiquitination [10]. Despite largely overlapping mRNA targets for ZC3H12A/Reg-1, ZC3H12B/Reg-2 and ZC3H12D/Reg-4 the phenotypes of Zc3h12a−/− and Zc3h12d−/− mice are different. Zc3h12a−/− mice develop severe systemic inflammation (including autoimmunity) manifested by an increase in immunoglobulin-producing plasma cells and the formation of granulomas. Infiltration of plasma cells to lungs, spleen, pancreas and lymph nodes, hyperimmunoglobulinemia of all immunoglobulin isotypes and increased production of IL-6 and IL-12p40 in response to TLR ligands were observed [1]. Surprisingly, whereas lack of Zc3h12a resulted in premature mice death at relatively young age (12 weeks), the Zc3h12d knock-out mice develop without visible pathology even though they synthesize increased amounts of IL-2, IL-6 and IL-10 [11]. The involvement of ZC3H12D in the regulation of inflammation is also suggested by its enrichment in inflamed organs such as spleen and lung as well as in lymph nodes [10] and by a sustained, upregulated levels of activated T cells [11]. In addition, experimental autoimmune encephalitis (EAE) induced in Zc3h12d−/− mice results in prolonged severe paralysis during the resolution phase and increased infiltration of Th17 cells [11]. No data is available on phenotype of mice lacking Zc3h12b.

Similarly to Zc3h12d−/−, Zc3h12c−/− mice develop without visible disorders, however they exhibit severe lymphadenopathy. Enlarged lymph nodes were particularly disrupted in B and T cell zones due to an impaired follicle and germinal center formation [12]. However, in contrast to Zc3h12a−/− mice, Zc3h12c−/− mice do not develop systemic autoimmunity. Interestingly, systemically increased interferon (IFN) signaling accompanied by enhanced STAT1 phosphorylation was described in Zc3h12c-deficient mice. ZC3H12C/Regnase-3 (Reg-3) binds to IFNγ mRNA but does not regulate its level. It was also shown that Reg-3 regulates Reg-1 level in a NYN/PIN domain-dependent manner. This observation is in agreement with the results showing increased amount of Reg-1 in Zc3h12c−/− macrophages [12]. As mentioned above the proteins from the ZC3H12/MCPIP/Regnase family share substrate specificity, for example they all regulate the turnover of IL-6 mRNA [1,6,7]. Surprisingly, changes in IL-6 level in the serum of Zc3h12c−/− mice in comparison to WT mice are not observed [12]. Since Reg-3 is highly expressed in the lung, liver, kidney and brain, whereas high levels of Reg-1 are observed in lymphoid tissues, differences in IL-6 regulation between Reg-1 and Reg-3 could result from the different tissue distribution of these proteins.

The data collected so far suggest an important role of the proteins from ZC3H12/MCPIP/Regnase family in the regulation of inflammation. While molecular mechanisms of Reg-1, Reg-2 and Reg-4 activity are relatively well described [3,6,7,11] the role of Reg-3 remains obscure.

A recent meta-analysis of genome-wide-association studies conducted by two independent groups, revealed that ZC3H12C/Reg-3 is among new psoriasis susceptibility loci [13,14]. Psoriasis is a chronic inflammatory skin disease, affecting about 2% of the global population.

In this study we have analyzed the mechanisms involved in Reg-3 function and examined a set of inflammation-related mRNAs as its possible targets, focusing on psoriasis, where Reg-3 may control disease-relevant mediators.

## 2. Results

### 2.1. Reg-3 Possesses Highly Conserved Domains Crucial for RNase Activity of other Members of the ZC3H12/MCPIP/Regnase Family

**Figure 1:**
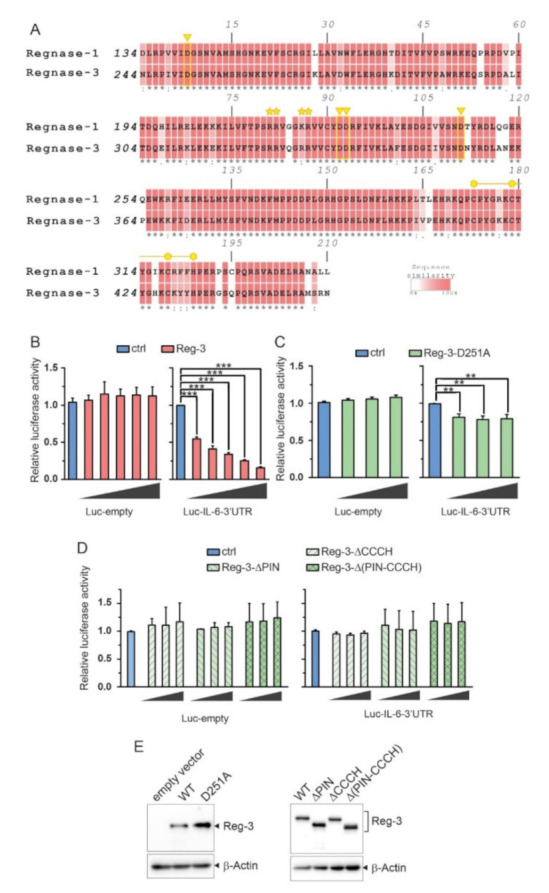
Reg-3 is involved in the 3′UTR-dependent destabilization of transcripts. (**A**) NYN/PIN and CCCH-zinc finger domain of Reg-3 contain all key residues essential for nuclease activity. Alignment of the NYN/PIN domain and CCCH-zinc finger of Reg-1 and Reg-3. The yellow triangles indicate the aspartic acid residues critical for binding of magnesium ion. The yellow line indicates the CCCH-zinc finger domain with three cysteine and one histidine residues indicated with hexagons. The asterisks denote the positively charged amino acid residues crucial for the binding of the negatively charged backbone of the substrate RNA. The alignment was performed using ClustalW2 [15] using the default settings and visualized using Adobe Illustrator 2020 (Adobe). (**B**–**E**) U251-MG cells were transfected with vectors coding for luciferase with attached *IL-6* 3′UTR (Luc-IL-6-3′UTR) or without any attached 3′UTR (Luc-empty) and varying amounts of Reg-3 wild-type (**B**), 10 to 160 ng/well in twofold increments) or Reg-3 D251A point mutant (**C**), 20 to 80 ng/well in twofold increments) or an empty control pcDNA3 vector (ctrl). (**D**) U251-MG cells were transfected with vectors coding for luciferase with attached IL-6 3′UTR (Luc-IL-6-3′UTR) or without any attached 3′UTR (Luc-empty) and varying amounts of Reg-3 muteins (ΔPIN, ΔCCCH or ΔPIN-CCCH, 20 to 80 ng/well in twofold increments) or an empty control pcDNA3 vector (ctrl). (**E**) The expression level of wild-type Reg-3 (WT) and specified muteins (D251A, ΔPIN, ΔCCCH or ΔPIN-CCCH) was examined by western blotting. Representative image of three independent experiments with similar results is shown. The presented graphs show the mean results of three independent experiments ±SD. The data were analyzed using one-way ANOVA (**B**,**C**) or two-way-ANOVA (**D**) with Bonferroni’s post-hoc test ([**] *p* < 0.01; [***] *p* < 0.001).

In order to examine the molecular function of Reg-3 we analyzed the degree of conservatism of two domains which are involved in the nucleolytic activity of the proteins from the ZC3H12/MCPIP/Regnase family. We aligned the sequence of the NYN/PIN domain and zinc finger domain of Reg-3 with that of Reg-1 (Figure 1A).

The overall conservativeness between Reg-1 and Reg-3 of NYN/PIN domain and CCCH domain is 84.4% and 76% respectively [5]. The crystallographic studies of Reg-1 revealed that four aspartic acid residues (D141, D225, D226, and D244) are crucial for magnesium ion binding and consequently nucleolytic activity of the protein [16]. All four residues are conserved in Reg-3 NYN/PIN domain supporting the idea of its catalytic function. The positively charged residues involved in the interaction with negatively charged transcripts are also conserved among Reg-1 and Reg-3. Additionally, a CCCH-type zinc finger domain is located in close proximity to the NYN/PIN domain which is important for RNase function including binding and cleavage of target RNA. Thus the conservation of residues crucial for RNase activity of Reg-1 is maintained in Reg-3.

### 2.2. The Importance of the NYN/PIN and CCCH Domains in Reg-3 Nucleolytic Activity

As a high level of Reg-3 is observed in the brain we have chosen human astrocytoma cell line, U251-MG, to study the biological function of this protein. We focused on the regulation of IL-6 mRNA turnover since the IL-6 transcript is recognized and degraded by all other members of the ZC3H12/MCPIP/Regnase family [1,6,7]. We used a luciferase reporter vector in which the sequence of the 3′UTR of IL-6 is attached to the luciferase coding sequence (CDS) (Figure 1B–D). The U251-MG cells were co-transfected with this vector (pLuc-IL-6-3′UTR) or control reporter vector without IL-6 3′UTR (pLuc-empty) and with increasing amounts of expression vector for Reg-3. Overexpression of Reg-3 was found to corelate with a decreased activity of luciferase encoded by pLuc-IL-6-3′UTR. The regulation was dose-dependent. Simultaneously, overexpression of Reg-3 did not influence the activity of luciferase encoded by the control vector, pLuc-empty (Figure 1B). To determine whether the NYN/PIN domain was important for Reg3-mediated regulation of IL-6 we have generated a mutant form of Reg-3 in which the crucial asparatic acid residue at position 251 engaged in catalytic center formation was replaced with alanine (D251A). U251-MG cells were co-transfected with reporter vector, pLuc-IL-6-3′UTR or pLuc-empty, and with increasing amounts of the expression vector encoding Reg-3 D251A mutein. Surprisingly, the mutant form of Reg-3 still affected the luciferase activity in the IL-6-3′UTR-dependent manner even though the effect is much weaker than the one observed for WT Reg-3 (Figure 1C). The reduced impact of Reg-3-D251A on luciferase level was not due to lower amount of the mutein (Figure 1E). These results are unexpected since the mutation of any of the crucial conserved aspartic acid in the NYN/PIN domain of Reg-1, Reg-2 and Reg-4 results in the abrogation of their nucleolytic function [1,6,7]. To study this further we have also mutated other three aspartic acid residues present in the catalytic center of Reg-3 (D335A, D336A, D354A) and observed similar results as for D251A mutation (data not shown). These results challenge the involvement of NYN/PIN domain in Reg-3-dependent transcript turn-over. To verify the role of conserved domains in this process we have generated deletion mutants of Reg-3, namely mutein without NYN/PIN domain (Reg-3-ΔPIN), without CCCH domain (Reg-3-ΔCCCH) and without both domains (Reg-3-ΔPIN-CCCH). The activity of luciferase in the lysates from cells co-transfected with increasing amounts of expression vectors for Reg-3-ΔPIN, Reg-3-ΔCCCH or Reg-3-ΔPIN-CCCH respectively and pLuc-IL-6-3′UTR or pLuc-empty was very similar to the activity of luciferase in the lysates transfected with the pLuc vectors alone (Figure 1D). Thus, the removal of any of these domains completely abolished Reg-3 involvement in the degradation of transcripts containing IL-6 3′UTR. The absence of luciferase regulation by muteins was not caused by their improper synthesis or lower level of expression (Figure 1E).

### 2.3. The Known Substrates of Reg-1, Reg-2 and Reg-4 Are also Substrates of Reg-3

**Figure 2:**
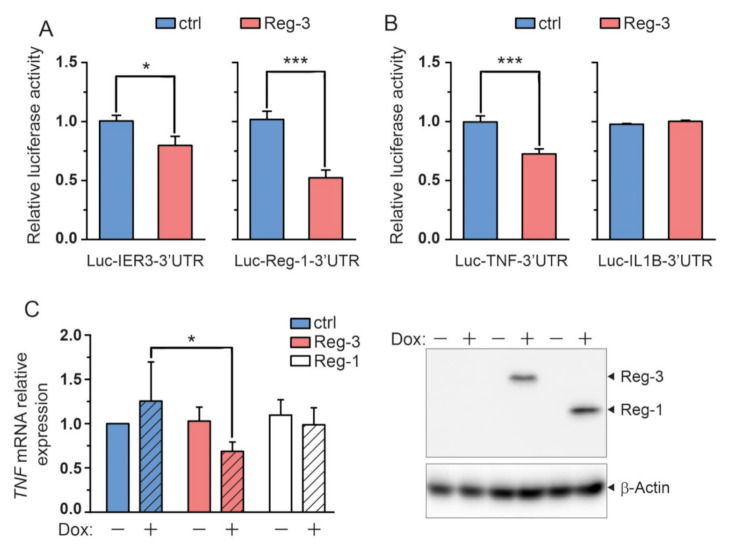
Regulation of known Reg-1 substrates and TNF by Reg-3. (**A**,**B**) U251-MG cells were transfected with vectors coding for luciferase with attached 3′UTRs of known Reg-1 substrates (*IER3*, *Reg-1* and *IL-1β*) and *TNF* (Luc-IER3-3′UTR, Luc-Reg-1-3′UTR, Luc-IL1B-3′UTR and Luc-TNF-3′UTR respectively) and Reg-3 or empty (ctrl) expression vectors (20 ng/well). (**C**) U251-MG cells with Dox-inducible expression of Reg-3, Reg-1 and luciferase (ctrl) were stimulated for 24 h with 10 ng/mL of Il-1β and then treated with Dox for 5 h or left untreated. The expression level of TNF mRNA was examined using RT-qPCR. The expression of Reg-3 and Reg-1 was verified by western blotting. Representative image of three independent experiments with similar results is shown. The presented graphs show the mean results of three independent experiments ±SD. The data were analyzed using one-way ANOVA (**A**,**B**) or two-way-ANOVA (**C**) with Bonferroni’s post-hoc test ([*] *p* < 0.01; [***] *p* < 0.001).

We and other groups have previously shown that Reg-1, Reg-2 and Reg-4 besides IL-6 mRNA recognize and degrade transcripts with the 3′UTRs of IER3 or Reg-1 [3,6,7]. The reporter vectors containing IER3 or Reg-1 3′UTR attached to the CDS of luciferase, pLuc-IER-3′UTR or pLuc-REG1-3′UTR respectively, were employed to analyze Reg-3 involvement in the turnover of mRNAs containing the investigated 3′UTRs. Overexpression of Reg-3 resulted in a decreased activity of luciferase in lysates from cells transfected with pLuc-IER-3′UTR or pLuc-REG1-3′UTR vectors in comparison to the control vector (pLuc-empty). Thus Reg-3 shares with all other members of the family inflammation-related targets. (Figure 2A).

### 2.4. Reg-3 Regulates the Level of TNF but Not IL-1 Transcript

Whereas the regulation of IL-6 mRNA level by the members of the ZC3H12/MCPIP/Regnase family is well documented, the involvement of these proteins in the control of the amount of two other main proinflammatory cytokines, namely IL-1β and TNF, is less explored. We have examined the regulation of transcripts containing *IL-1β* or *TNF* 3′UTRs by Reg-3. The U251-MG cells were co-transfected with pLuc-IL-1-3′UTR or pLuc-TNF-3′UTR and expression vector for Reg-3. The addition of the 3′UTR fragment of *TNF* to *luc* mRNA (pLuc-TNF-3′UTR) results in a Reg-3-dependent decrease in luciferase activity. In contrast, addition of *IL-1β* 3′UTR fragment to *luc* mRNA (pLuc-IL-1-3′UTR) does not influence the level of luciferase in the presence of Reg-3 (Figure 2B). To further confirm the involvement of Reg-3 in regulation of the TNF levels we have explored the regulation of endogenous TNF transcript by Reg-3. We have employed the Sleeping Beauty transposon system (SB) enabling an integration of the transgene into the genome and its inducible expression. This system provides limited number of integrated transgene cassettes and thus does not activate or overload specific biological pathways [17]. U251-MG cells were modified using the SB system allowing for doxycycline-inducible expression of Reg-3. To induce the synthesis of endogenous TNF, SB-modified U251-MG cells were stimulated with IL-1β for 24h and then treated for 5h with doxycycline (Dox). Dox-induced expression of Reg-3 results in the downregulation of TNF transcript levels. In contrast, modification of U251-MG cells by the SB transposon system encoding Reg-1 under the control of the same Dox-inducible promoter does not influence the level of endogenous TNF mRNA (Figure 2C). The amount of TNF transcript in SB-Reg-1-modified cells treated with Dox was very similar to the control (not treated) cells indicating that Reg-1 does not influence TNF mRNA levels. The observed differences in the pattern of the regulation of endogenous TNF mRNA level by Reg-1 and Reg-3 were not due to differences in their concentration. Following Dox treatment both Reg-3 and Reg-1 were expressed at approximately the same levels (Figure 2C). We concluded that Reg-3 in contrast to Reg-1 is involved in the control of cellular TNF level and does not control the level of IL-1β transcript.

### 2.5. Cellular Localization of Reg-3

**Figure 3:**
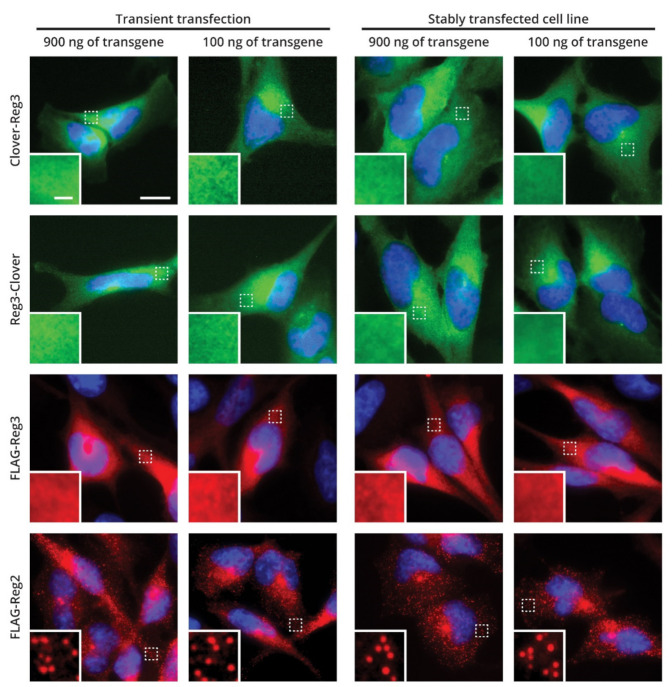
Reg-3 is a protein diffusely localized in the cytoplasm. Transiently (**left** panels) or stably (**right** panels) transfected U251-MG cells with indicated amounts of transgenes used per 1 µg of total DNA used for transfection (Clover-Reg-3, Reg-3-Clover, FLAG-Reg-3 or FLAG-Reg-2) were fixed and visualized under fluorescent microscope. The DNA was counterstained using DAPI (blue). Scale bars: main images: 10 µm, zooms: 1 µm.

Our previous results show that all other members of the ZC3H12/MCPIP/Regnase family are localized in the cytoplasm, but not in the nucleus, and form small, granule-like structures [6,7]. We generated recombinant Reg-3 tagged with Clover fluorescent protein at its N-terminus and investigated the localization of this protein in U251-MG cells. Clover-Reg-3 is detected in the cytoplasm of transfected cells. However, we observed its even distribution in the cytoplasm and absence of granule like structures (Figure 3). We speculated that the lack of granules can result from transient transfection of the cells and related possible artefacts so we have analyzed the pattern of Clover-Reg-3 localization in stably transfected cells. To generate stably transfected cell lines we have used the described above SB transposon system. However, we have obtained exactly the same expression pattern as observed in the transiently transfected cells. The amount of the Reg-3-containing transposon vector used for stable cell line preparation (900 ng or 100 ng) also did not change the pattern of Reg-3 cellular localization. These results did not exclude the unintended effect of the tag on the Reg-3 N-terminus, which could potentially affect subcellular localization of Reg-3. Thus, we attached the tag to the C-terminus of Reg-3. Still, this modification did not change the pattern of Reg-3 distribution in the cells. Likewise, the replacement of the protein tag with a smaller one (Clover to FLAG) also did not change the localization of Reg-3 in the cells. In contrast, FLAG-Reg-2 is localized in small granule like structures in both transiently and stably transfected cells (Figure 3). We conclude that Reg-3 differs from other members of the ZC3H12/MCPIP/Regnase family in subcellular distribution pattern.

### 2.6. The Role of Reg-3 in Psoriasis

Our finding that Reg-3 regulates levels of TNF (Figure 3), a cytokine strongly implicated in development of psoriasis flares, suggested that Reg-3 might play a controlling role in the development/severity of this disease. Psoriasis is characterized by skin infiltration with neutrophils and other leukocytes, as well as keratinocyte hyperproliferation and dysfunction.

**Figure 4:**
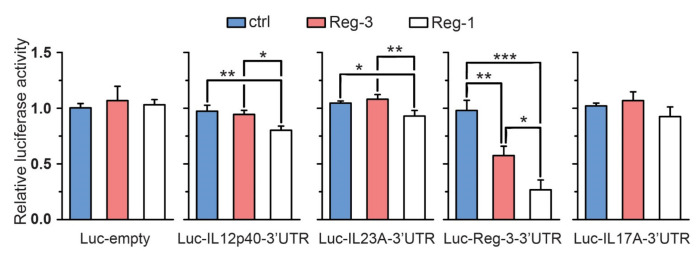
Regulation of psoriasis-related transcripts by Reg-1 and Reg-3. Cells were transfected with vectors coding for luciferase with attached 3′UTRs of mRNAs related to psoriasis (*IL-12p40, IL-17A, IL-23A,* and *Reg-3*) or empty control plasmid (Luc-IL12p40-3′UTR, Luc-IL17A-3′UTR, Luc-IL23A-3′UTR, Luc-Reg-3-3′UTR and Luc-empty respectively) and Reg-3, Reg1 or empty (ctrl) expression vectors (20 ng/well). The presented graphs show the mean results of three independent experiments ±SD. The data were analyzed using one-way ANOVA with Bonferroni’s post-hoc test ([*] *p* < 0.5; [**] *p* < 0.01; [***] *p* < 0.001).

Along TNF, other cytokines such as IL-17 and IL-23 are considered key drivers of chronic inflammation in psoriasis. The role of these proinflammatory factors in psoriasis is highlighted by targeted therapy against IL-17 and/or IL-23 that has proven successful in the treatment of psoriasis [18]. To determine whether Reg-3 destabilizes transcripts for these cytokines we cloned 3′UTRs of IL-17A, IL-23A, IL-12p40 (a common subunit for IL-23beta and IL-12) and Reg-3 itself. The UTR sequences were attached to the CDS of luciferase and the obtained reporter vectors (pLuc-IL17-3′UTR, pLuc-IL12p40-3′UTR, pLuc-IL23A-3′UTR and pLuc-Reg3-3′UTR respectively) were used for co-transfection of U251-MG cells together with expression vectors for Reg-3 or Reg-1. So far published results indicate that IL-17A 3′UTR is recognized by Reg-1 but not by Reg-2 or Reg-4. Attachment of IL-17A 3′UTR to the CDS of luciferase did not influence the turnover of the recombinant reporter transcript neither by Reg-3 (Figure 4) nor by Reg-1. Our results confirm the already described involvement of Reg-1 in the regulation of IL-12p40 [1] and show a Reg-1-dependent down regulation of luciferase mRNA with IL-23A 3′UTR. In contrast to Reg-1, Reg-3 did not affect the level of recombinant mRNAs with attached IL-12p40 or IL-23A 3′UTR (Figure 4). Interestingly, Reg-1 and to a smaller extent Reg-3 significantly reduced the levels of luc mRNA with Reg-3 3′UTR (Figure 4), suggesting that Reg-3 levels are tightly controlled, primarily by Reg-1.

**Figure 5:**
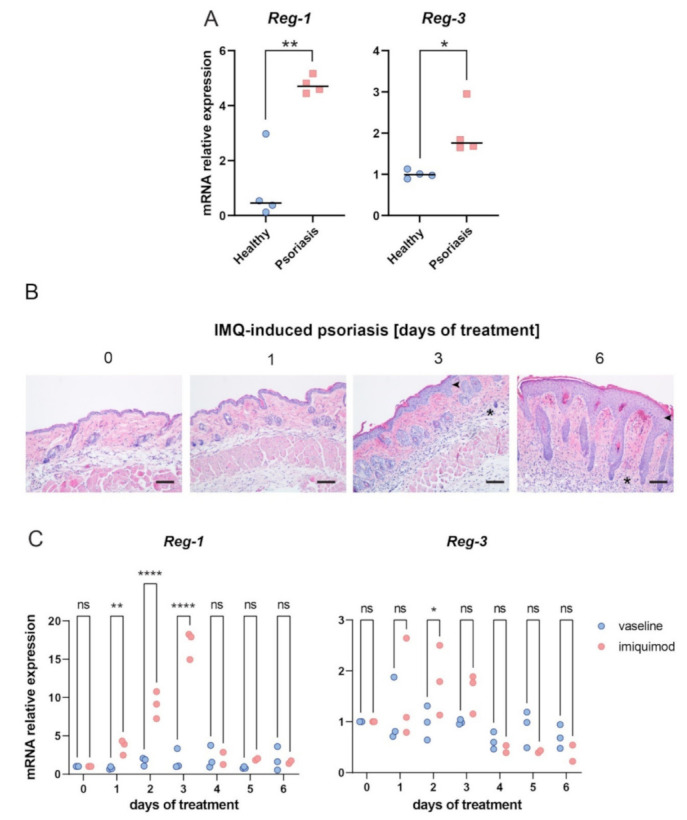
Reg-1 and Reg-3 transcript levels are increased in lesional skin of patients with psoriasis and in IMQ-driven experimental model of psoriasis. (**A**) Reg-1 and Reg-3 mRNA expression was measured in the skin biopsies from the indicated human donors by RT-qPCR. The expression data of the indicated genes was normalized to *GAPDH* and presented relative to healthy donors as data points and the mean value in each group (horizontal line), *n* = 4 different donors. (**B**) Mice were subjected to IMQ-based experimental model of psoriasis. Skin alterations, including increased epidermal thickness (arrowhead) and leukocyte infiltration (asterisk) were detected by histology at the indicated time points. Scale bars = 50 μm. Data sets are representative for at least 5 mice. (**C**) Reg1 and Reg-3 mRNA expression was measured at the indicated time points in IMQ-treated skin of WT mice (red data points). Vaseline-treated skin served as negative control (blue data points). The expression data of the indicated genes was normalized to *Eef2* and presented relative to vaseline-treated mice. *n* = 3 independent experiments. Significance is indicated by asterisk(s); * *p* < 0.05, ** *p* < 0.01, **** *p* < 0.0001, ns = non significant by two-way-ANOVA with Bonferroni post hoc test.

To determine whether Reg-3 is differentially regulated in psoriasis, we analyzed the expression of Reg-3 and, for comparison, Reg-1 in skin samples derived from psoriasis patients and age- and sex-matched healthy donors [19]. RT-qPCR revealed significant upregulation of Reg-1 (on average 5x) and to a lesser extent Reg-3, (approx. 2×) in psoriasis individuals compared to heathy controls (Figure 5A).

Psoriasis is a complex disease that evolves in time, and Reg-3 as well as Reg-1 might be associated with specific stage(s) of the disease. We employed experimental, imiquimod (IMQ)-based model of psoriasis [20], that allowed us to analyze psoriasis-associated expression levels of Reg-3 as well as Reg-1 in a kinetic-dependent fashion. Vaseline-treated skin was used as a control. Histology of skin samples collected at day 0, 1, 3 and 6 after application of IMQ confirmed psoriasis-like changes in this model, such as skin thickening and robust skin infiltration (Figure 5B).

In IMQ-treated mice, we observed a tendency for an increased (on average 2–2.5 times higher) Reg-3 expression at early stages of skin challenge (day 1–3), that reached a statistical significance at day 2, but not at latter stages (day 4–6), (Figure 5C). Reg-1 was upregulated with similar kinetics, but to much higher levels compared with Reg-3. Approximately 10 times more of Reg-1 was found to be expressed at day 3 of IMQ-treated mice compared with vaseline-treated controls (Figure 5C). Together, these data suggest rapid upregulation of Reg-1 and to a lower extent of Reg-3 expression levels in psoriasis-like pathology.

**Figure 6:**
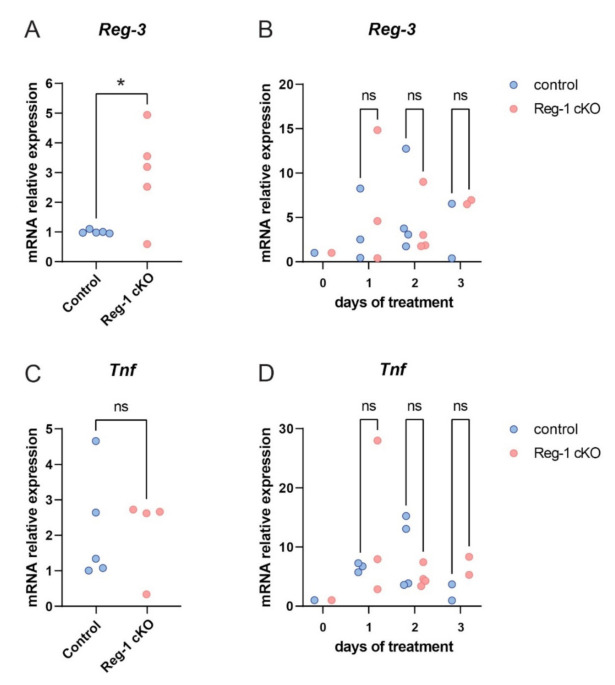
Regulation of *Reg-3* and *TNF* in the skin in the context of keratinocyte Reg-1 deficiency. Reg-1 cKO mice and their littermate controls were left untreated (**A**,**C**) or were subjected to IMQ treatment for upto 3 days (**B**,**D**), followed by RT-qPCR analysis of the indicated genes. The expression data was normalized to *cyclophilin* and presented relative to control mice (**A**,**C**) or relative to time 0 (**B**,**D**). *n* = 2–5 independent experiments. Significance is indicated by asterisk; * *p* < 0.05, ns = non significant by *t*-test (**A**,**C**) or two-way-ANOVA with Bonferroni post hoc test (**B**,**D**).

Psoriasis is associated with dramatic alterations in proliferation and differentiation of keratinocytes, a dominant cell type in epidermis [18,21]. Given that Reg-3 mRNA is degraded by Reg-1 in vitro (Figure 4), more robustly upregulated Reg-1 mRNA levels compared to Reg-3 transcript levels in the skin of IMQ-treated mice suggested that Reg-3 might be constantly or transiently downregulated by Reg-1, especially in keratinocytes, which are functionally strongly dependent on Reg-1 [22]. Indeed, we found that keratinocyte-specific Reg-1 knockout mice (Reg1 cKO) [22] demonstrated significantly elevated Reg-3 levels at baseline compared to control mice (Figure 6A).

Likewise, in IMQ-model, Reg-3 showed an overall tendency for a higher expression in the absence of Reg-1 in keratinocytes (Figure 6B). Of note, expression of TNF, a postulated target of Reg-3 was not significantly changed at baseline in Reg-1 cKO mice (Figure 6C) but demonstrated a similar to Reg-3 pattern of regulation in the skin of control and Reg-1 cKO mice in response to IMQ treatment (Figure 6D). This correlative presence of Reg-3 and TNF in psoriatic skin further suggests that Reg-3 may serve as a regulator of TNF levels in psoriasis.

**Figure 7:**
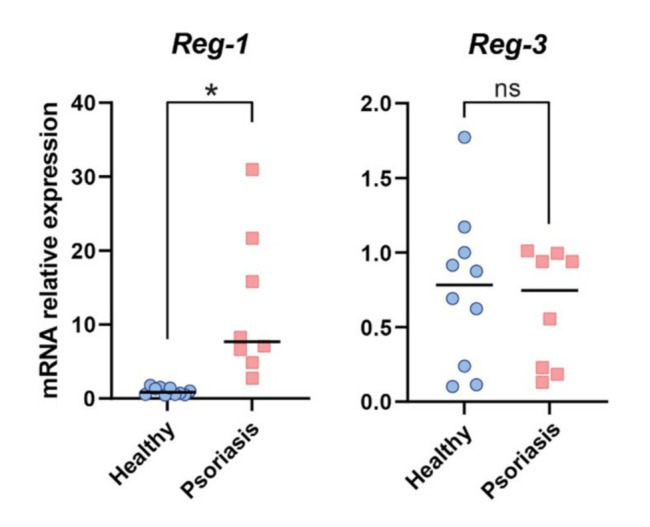
Expression of *Reg-1* and *Reg-3* is differently regulated in human epidermis in association with psoriasis. Reg-1 mRNA expression and Reg-3 mRNA expression was measured in epidermis derived from the skin biopsies of the indicated human donors, by RT-qPCR. The expression data of the indicated genes was normalized to *GAPDH* and presented relative to healthy donors as data points and the mean value in each group (horizontal line), *n* = 8 different donors. Significance is indicated by asterisk(s); * *p* < 0.05, ns = non significant by ANOVA followed by a Bonferroni post hoc test.

We next performed RT-qPCR analysis on epidermal samples derived from lesional skin of psoriasis individuals and healthy donors to determine whether Reg-3 is differentially regulated in epidermis of psoriasis patients. As shown in Figure 7, Reg-3 was not significantly upregulated in epidermis of psoriasis patients as compared to healthy controls. In contrast, Reg-1 was significantly increased. Together, these data suggest that both Regnases are expressed in keratinocytes, but only Reg-1 is differentially regulated in epidermis in association with psoriasis.

Nevertheless, substantial differences in Reg-3 mRNA levels were noted in epidermis of both healthy donors and patients with psoriasis as well as in Reg-1 in individuals suffering from psoriasis (Figure 7).

**Figure 8:**
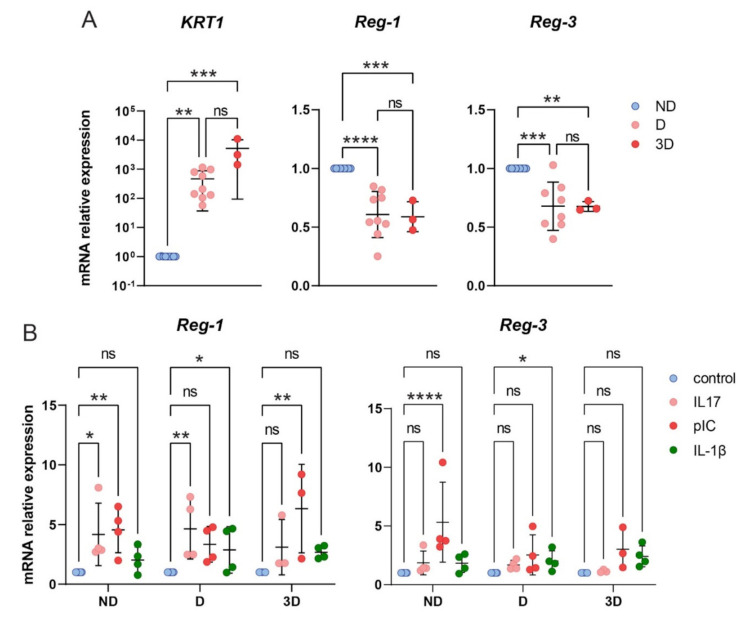
*Reg-3* is expressed in human keratinocytes in proliferation-dependent manner. (**A**) Human keratinocytes were grown in the indicated cultures and subjected to RNA isolation followed by RT-qPCR. The expression data of the indicated genes was normalized to *GAPDH* and presented relative to undifferentiated keratinocytes (ND) as data points and the mean value in each group (bars), *n* = 3–9 independent donors. (**B**) Poly(I:C), an analog of viral dsRNA increases Reg-3 expression in mitotically active human keratinocytes. Human keratinocytes grown in the indicates cultures were treated with 200 ng/mL IL-17, 10 ng/mL IL-1β and 10 µg/mL poly(I:C) for 24 h. Total RNA was subjected to RT-qPCR. Relative expression of Reg-1 or Reg-3 in stimulated cells over control is shown as the mean from three independent experiments. * *p* < 0.05, ** *p* < 0.01, *** *p* < 0.001, **** *p* < 0.0001, ns = not significant by ANOVA followed by a Bonferroni post hoc test.

Potential changes in Reg-3 mRNA levels may be masked by heterogeneity of keratinocytes that may differ in Reg-3 expression based on proliferation/differentiation status. To determine whether Reg-1 and Reg-3 expression is associated with differentiating or proliferating keratinocytes, we next employed 2D and 3D cultures of human keratinocytes. Keratinocytes were grown under conditions that support mostly proliferation (nondifferentiated 2D cultures; ND), partial differentiation (partially differentiated 2D cultures, D) and differentiated organotypic-like 3D cultures (3D) [23] (Figure 8). Expression of keratin-1, a marker of differentiated keratinocytes showed the expected changes between ND, D and 3D models (Figure 8). Notably, either Reg-1 or Reg-3 were mostly expressed at mRNA levels in proliferating keratinocytes, suggesting that mitotically active basal cells are the main source of these Regnase transcripts in epidermis. Nevertheless, when Reg-1 and Reg-3 expression levels were analyzed in parallel in the proliferating keratinocytes derived from the same individuals by RT-qPCR, Reg-3 mRNA levels were from 2 to 23 times lower compared to Reg-1 mRNA levels (*n* = 6 donors, data not shown).

To better understand the substantial heterogeneity in Reg-3 mRNA levels in keratinocytes, we stimulated ND, D and/or 3D cultures with poly(I:C), IL-17, IL1β, TNF and IL6, strong drivers of keratinocyte responses and/or potential targets of Regnase-3 activity in keratinocytes [24,25] and Figure 1 and Figure 2. Neither TNF nor IL6 significantly altered Reg-1 and Reg-3 transcript levels in ND and D cultures (data not shown). Il-1β was the only stimulus, which upregulated levels of both Reg-1 and Reg-3 only in D cultures (Figure 8). Whereas IL-17 was ineffective in regulation of Reg-3 levels in either proliferating or differentiating keratinocytes, poly(I:C) was found to significantly increase Reg-3 levels in proliferating keratinocytes (Figure 8). In contrast, Reg-1 was responsive to both IL-17 and poly(I:C). IL-17 triggered expression of Reg-1 mostly in ND and D, and poly(I:C) in 3D keratinocytes (Figure 8). Taken together, these findings highlight the potential differences in expression patterns and regulation pathways between Reg-1 and Reg-3 in the skin.

## 3. Discussion

The molecular mechanisms of the nucleolytic properties of the three members of the ZC3H12/MCPIP/Regnase family of proteins, namely ZC3H12A/MCPIP1/Reg-1, ZC3H12B/MCPIP2/Reg-2 and ZC3H12D/MCPIP4/Reg-4 were recently described [3,6,7,11]. Moreover, certain targets recognized by them were identified. Since the last member of this family, ZC3H12C/MCPIP3/Reg-3, was only partially investigated so far, we have analyzed the molecular properties of this protein and its cellular targets. We have confirmed the importance of NYN/PIN and CCCH domains in its biological function, however, in contrast to other family members, mutation of crucial aspartic acid residues in the catalytic domain of Reg-3 does not result in the complete inactivation of Reg-3 ability to degrade target mRNAs. The rate of the turnover of recombinant transcript with IL-6-3′UTR in the presence of the muteins is inhibited when compared to the wild-type protein but still present. One possible explanation of this observation could be that Reg-3 itself has low RNase activity and it participates in transcript degradation by forming the complexes with other RNases. Such scenario would also explain why the removal of the whole NYN/PIN or CCCH domains abolishes Reg-3-dependent down-regulation of investigated transcripts. Besides the implication of the CCCH domain in the interaction with targets, both domains could be involved in the interaction with other proteins, including RNases. Till now the direct interaction of Reg-1 and ZC3H12D/Reg-4 on IL-6 mRNA was described [26]. The fragments including amino acids 301–457 of Reg-1 and 259-356 of Reg-4, thus the CCCH-domains of both proteins, were revealed as responsible for this interaction. Another difference regarding Reg-3 in comparison to other members of the ZC3H12/MCPIP/Regnase family is the pattern of its cellular localization. In contrast to Reg-1, -2, and -4 [6,7,26,27] Reg-3 does not form granule-like structures, instead this protein is evenly distributed in the cytoplasm. This is not typical for proteins that participate in the turnover of transcripts however such proteins as G3BP exhibit similar pattern of localization. G3BP is a major protein component of granular ribonucleoprotein complexes called stress granules (SGs) which form in the cytoplasm upon exposure of cells to stress. In steady-state conditions G3BP is diffusively distributed in the cytoplasm, however forms granules upon stress [28]. The verification of whether Reg-3 is able to form granular structures under stress or after stimulation with certain agents needs further studies. Regardless of the differences between Reg-3 and other ZC3H12/MCPIP/Regnase family members, we also found that Reg-3 shares several mRNA targets with Reg-1, Reg-2 and Reg-4, including IL-6, IER-3 and Reg-1 3′UTRs. In addition, we have shown that Reg-3 regulates its own transcript. This last observation suggests a regulatory loop within the ZC3H12/MCPIP/Regnase family. We have discovered that not only Reg-3 regulates its own transcript and the transcript of Reg-1 but also Reg-1 regulates Reg-3 (and also its own transcript [29]. Among other notable differences between Reg-3 and other members of the family is the regulation of TNF mRNA. Our data revealed that Reg-3 is the only member of the ZC3H12/MCPIP/Regnase family which has such properties. Although, Mino et al. have shown that Reg-1 interacts with TNF 3′UTR [3] other studies, including our results, do not support these findings. In the first report describing the role of Reg-1 in the control of immune response published by Akira’s group, the results obtained using various methods prove that endogenous TNF level is not regulated by Reg-1 [1]. Stimulation of peritoneal macrophages with different TLR-substrates such as MALP-2, poly(I:C), LPS, R-848 and CpG-DNA for 24 h followed by measurement of TNF level in the culture medium does not show an increase in the amount of TNF in the medium collected from Zc3h12a−/− samples compared to Zc3h12a+/+. Also the detailed analysis of the kinetics of LPS-induced TNF transcript turnover in macrophages from Zc3h12a−/− and Zc3h12a+/+ mice reveals exactly the same half-life of TNF mRNA in the investigated cells [1]. Our data indicate that Reg-3 not only interacts with transcripts with attached TNF 3′UTR but is also involved in the regulation of endogenous TNF transcript levels.

The regulation of TNF half-life by Reg-3 might be of relevance to psoriasis. Reg-3 is among the recently identified genetic risk variants associated with this disorder [13,14]. Keratinocytes and leukocytes both contribute to pathogenic inflammatory cycle in psoriasis with a central role of TNF/IL-23/IL-17 in chronic skin inflammation [18,21]. Whereas Reg-1 was recently reported to negatively regulate IL-23/IL-17 transcript levels at early stages of IMQ-driven experimental psoriasis [30] our data suggest that Reg-3 might also reduce pathogenic inflammation by directly controlling TNF turnover in psoriasis. The involvement of Reg-1 and Reg-3 in psoriasis pathophysiology is also supported by differential expression of both RNases in skin of psoriasis patients (Figure 5). However, spatiotemporal impact of Reg-1 and Reg-3 on skin alterations is likely to be different, as suggested by strong upregulation of Reg-1 but not Reg-3 in epidermis of psoriasis patients (Figure 7). Keratinocytes, the major cellular component of epidermis, are highly responsive to TNF, and this cytokine induces key inflammatory programs in psoriatic keratinocytes [25]. However, epidermal keratinocytes, might not be the main source or site of Reg-3 activity in lesional skin of patients with psoriasis. Dermis located myeloid cells such as macrophages that are known to highly expressed Reg-3 [12] might be an additional source of this RNAse in chronically inflamed skin. On the other hand, our data indicate that Reg-3 is produced in epidermal keratinocytes, especially in mitotically active cells. Since Reg-1 strongly limits Reg-3 levels (Figure 4), lack of Reg-3 upregulation in psoriatic epidermis, can result from degradation of Reg-3 transcripts by dominant Reg-1 that is more abundantly expressed in psoriatic keratinocytes. This is supported by our data showing elevated levels of Reg-3 mRNA in epidermis when expression of Reg-1 is ablated (Figure 6). Among other changes (in subcellular distribution or function) reported here for Reg-1 and Reg-3, our data also point at differences in the regulation of expression of these RNases in keratinocytes. For example, in line with previous report, Reg-1 expression is induced in keratinocytes by IL-17 [31]. In contrast, Reg-3 was found not to be significantly regulated in keratinocytes by IL-17, but induced in the proliferating cells in response to immunostimulating factor poly(I:C), an analog of dsRNA. Poly(I:C) mimics viral infection and drives type I interferon (IFNI) responses. TNF is known to be negatively regulated by IFNI in chronic inflammatory diseases, including psoriasis [32]. TNF inhibition with anti-TNF therapy leads to an ongoing IFNI-mediated inflammation in psoriasis [21]. Given that poly(I:C), upregulates Reg-3 levels in keratinocytes, our studies suggest that signaling loops leading to IFNI expression may contribute to restricting TNF levels partly through Reg-3-mediated alteration of the stability of TNF transcripts. Taken together, our results reveal unique features of Reg-3 compared to the other family members, including the inability of NYN/PIN-domain point mutations to inactivate Reg-3 activity and the cellular localization pattern of Reg-3. Reg-3 shares substrate specificity with other family members, but at the same time recognizes its unique targets, such as TNF mRNA. By controlling TNF and Reg-1 transcripts, Reg-3 can be involved in the pathophysiology of psoriasis. Thus, not only Reg-1 but also Reg-3 and the interplay between them is likely to have an impact on the progression of this disease.

## 4. Materials and Methods

### 4.1. Cell Culture

U251-MG cell line was maintained in DMEM with 4.5 g/L D-glucose (Lonza, Walkersville, MD, USA) supplemented with 10% fetal bovine serum. The Sleeping Beauty transposon-based cell lines with doxycycline inducible transgene expression were cultured in media supplemented with 10% tetracycline-free fetal bovine serum (Biowest, Nuaillé, France) and 2 μg/mL puromycin (Invivogen, San Diego, CA, USA). In stimulation experiments for RT-qPCR the cells were grown in reduced serum medium (0.5% FBS) 24 h prior to stimulation. Cell lines were maintained at 37 °C in a humidified atmosphere with 5% CO_2_. All cell cultures were examined on a regular basis for mycoplasma contamination using PCR [33].

### 4.2. Clinical Material

All human studies were performed in compliance with ethical protocols KBET/44/B/2011 and KBET/87/B/2014 approved by the Jagiellonian University Institutional Bioethics Committee. Declaration of Helsinki protocols were followed. In total, nine psoriasis patients (age 39 ± 18; F:M, 5:4) and 19 donors without any dermatological diseases (age 49 ± 28; F:M, 10:9), were enrolled in the study [19,23,34]. The severity of the psoriatic skin lesions was assessed according to the Psoriasis Area Severity Index score (PASI) (minimum, 0 points; maximum, 72 points) and ranged from 12 to 26. Skin biopsies were directly subjected to RT-qPCR or to isolation of epidermis and/or keratinocytes, followed by RT-qPCR.

### 4.3. Isolation of Epidermis and Cell Culture

Normal human keratinocytes were isolated from excess skin from donors obtained at the time of different surgery procedures, including cosmetic surgery for mole removal or plastic surgery. Skin biopsies (from either normal or psoriasis donors) were rinsed in PBS supplemented with penicillin (5000 U/mL)—streptomycin (5 mg/mL) (all from Sigma-Aldrich, St. Louis, MO, USA). After washing, the biopsy was placed in PBS containing dispase (10 mg/mL, Gibco, Gaithersburg, MD, USA) for 16 h at 4 °C. Next, the epidermis was separated from the dermis with forceps and subjected to RT-qPCR or treated with trypsin solution (Gibco) to isolate epidermal cells. Cells were cultured in DMEM medium with 10% FBS for 8 h followed by serum free KGM-Gold medium (Lonza, Walkersville, MD, USA) to generate passage 1 cells that were subjected to 2D or 3D cultures. To generate undifferentiated 2D cells, the keratinocytes were plated at density of 1 × 10^5^ cells per well on 24-well plate and cultured in CnT Prime Epithelial Culture Medium (CellnTec, Bern, Switzerland) for 3 days before stimulation. To generate partly differentiated 2D cells, normal human keratinocytes were plated at density of 1 × 105 cells per well on 24-well plate and cultured in CnT Prime 3D Barrier Medium (CellnTec, Bern, Switzerland) for 3 days before stimulation. Organotypic-like 3D keratinocyte cultures were generated as previously described [23] with minor modification. Briefly, keratinocytes were plated at density of 1.75 × 105 cells per well on 12 mm diameter, 0.4 µm pore size permeable inserts (Millipore) in CnT Prime Epithelial Culture Medium (CellnTec, Bern, Switzerland). Cells were cultured at 37 °C in presence of 5% CO_2_ for 3 days. Stratified structure of cultured keratinocytes was generated on air-liquid phase in CnT Prime 3D Barrier Medium (CellnTec, Bern, Switzerland) for 11 days. Cells were than stimulated with 200 ng/mL IL17 (R&D Systems, Minneapolis, MN, USA), 10 ng/mL IL1β (Peprotech, Rocky Hill, NJ, USA) or 10 µg/mL poly(I:C) (Invivogen, San Diego, CA, USA) for 24 h.

### 4.4. Experimental Psoriasis

C57BL6 mice (7–12 weeks old), Reg-1 keratinocyte deficient Reg-1 cKO and their littermate controls Reg-1^loxP/loxP^ all on C57BL6 background [22] were housed under pathogen-free conditions in the animal facility at the Faculty of Biochemistry, Biophysics and Biotechnology of the Jagiellonian University. Reg-1cKO mice were generated by breading male Krt14^Cre^ mice with female Reg-1^loxP/loxP^ mice, as previously described [22]. All animal studies were approved by, and conducted in compliance with, the guidelines of the Second Local Ethical Committee on Animal Testing at the Institute of Pharmacology Polish Academy of Sciences in Krakow (approvals # 298/2017 and # 118/2021). An Imiquimod (IMQ) model of psoriasis was induced as previously described [35] with minor modifications. Briefly, mice were treated twice daily for up to 6 days with 15 mg of Aldara™ cream (Meda AB, Solna, Sweden),) on shaved and depilated back skin. At indicated time points, skin sections comprising the center of treatment were placed in stayRNA (A&A Biotechnology, Gdańsk, Poland) and analyzed by RT-qPCR.

### 4.5. Histology

Mouse skin was fixed in PBS-buffered 4% formaldehyde and embedded in paraffin. 10 μm thick skin sections were stained with haematoxylin and eosin (Thermo Scientific, Waltham, MA, USA).

### 4.6. Cell Line Transfections

U251-MG cells were transfected with plasmid DNA using PEI MAX 40K (Polysciences, Warrington, PA, USA) or Lipofectaimne 2000 (Invitrogen, Carlsbad, CA, USA) with a 3:1 (w:w) ratio. Concisely, appropriate amounts of DNA and transfection reagent (PEI MAX 40K or Lipofectamine 2000) were diluted in Opti-MEM medium (Gibco, Grand Island, NY, USA), vortexed and centrifuged briefly. The Opti-MEM diluted transfection reagent was transferred to the diluted DNA, the solutions were mixed by vortexing, centrifuged briefly and incubated at room temperature for 20 min. After incubation the solution was applied onto the cells dropwise and the cells were incubated with the transfection solution for 4 h, then the solution was removed and replaced with fresh culture medium.

### 4.7. Establishment of U251-MG Cell Lines with Doxycycline Inducible Expression of Regnase-3 and Regnase-1

U251-MG cells were seeded in 12-well plates and the following day the cells were transfected with 900 ng of pSBtet-GP-Regnase-3, pSBtet-GP-Regnase-1 or pSBtet-GP vectors and 100 ng of the pCMV(CAT)T7-SB100 vector. 24 h later the cells were trypsinized and 1/5th of the cells was transferred into 6-well plates and cells with transposon integration were selected with puromycin (2 μg/mL, Invivogen) for 1 week. pCMV(CAT)T7-SB100 was a gift from Zsuzsanna Izsvak (Addgene plasmid # 34879; http://n2t.net/addgene:34879, accessed on 20 June 2021; RRID:Addgene_34879) [36].

### 4.8. Plasmid Construction

Human Regnase-3 (ZC3H12C, NM_033390.2) coding sequence was amplified with the following primers, 5′-TAGCGGCCGCCATGCCGGGTGGCGGCTCC-3′ and ATATCTCGAGTTAATAACCCAGCTGGGATTTCTCC, using the Q5 Hot Start High-Fidelity 2X Master Mix with human cDNA mix as a template. The appropriate PCR product was cloned into the pcDNA5/FRT/TO-SF-ZC3H12B vector described previously [7] by substitution of ZC3H12B coding sequence (CDS) with Regnase-3 CDS using traditional cloning techniques creating the pcDNA5-SF-Regnase-3 vector. Construct for Regnase-3 mutein with point mutation in the PIN domain (D251A) were obtained using QuickChange Site Directed Mutagenesis Kit (Agilent, Cedar Creek, TX, USA) and the following primers: 5′-AATCTGAGACCAATAGTTATTGCTGGCAGCAATGTG GC-3′ and 5′-GCCACATTGCTGCCAGCAATAACTATTGGTCTCAGATT-3′. Constructs for Regnase-3 muteins lacking the PIN and/or the CCCH domains were obtained using Q5 Site Directed Mutagenesis Kit (NEB, Ipswich, MA, USA) and the following primers: 5′-ATTTTCACCATCATCTGTTACAATCTCTTG-3′ and 5′-ATTGTTCCTGAACACAAAA AGCAG-3′ for PIN deletion and 5′-AGGCTGCTTTTTGTGTTCAGGAAC-3′ and 5′-CCCGAAAGGGGCAGTCAG-3′ for CCCH deletion or the former PIN primer and the latter CCCH primer for deletion of both domains. Construct for doxycycline (Dox) inducible expression of Regnase-3 in the Sleeping Beauty (SB) transposon system (pSBtet-GP-Reg-3) was prepared through substitution of the luciferase CDS with Regnase-3 CDS by ligation of SfiI (EurX, Gdańsk, Poland) digested PCR product into SfiI linearized pSBtet-GP vector. The PCR product was obtained on the template of pcDNA5-SF-Regnase-3 using the following primers: 5′-AGGCCTGACAGGCCTTAATAACCCA GCTGGGATTTCTCC-3′ and 5′-AGGCCTCTGAGGCCACCCACCATGGCAAGCTGG AGC-3′. pSBtet-GP was a gift from Eric Kowarz (Addgene plasmid # 60495; http://n2t.net/addgene:60495, accessed on 19 June 2021; RRID:Addgene_60495) [37]. Expression plasmid for Dox-inducible Regnase-1 (ZC3H12A) expression in the SB transposon system was obtained as described for Regnase-3 but with the following primers: 5′-GGCCTCTGAGGCCCACCATGAGTGGCCC-3′ and 5′-GGCCTGACAGGCCTCAA TGGTGATGGTGATGATGACCG-3′. Vectors for Dox inducible Regnase-3 expression N-terminally fused with Clover fluorescent protein were prepared by traditional cloning techniques of PCR amplified Regnase-3 and Clover products into pSBtet-Pur plasmid. The following primers were used amplification of Regnase-3: 5′-AGGCCTCTGAGGC CCCTGCAGGCACCATGCCGGGTGGCGGCTCC-3′ and 5′-AGGCCTGACAGGCCG GCGGCCGCATAACCCAGCTGGGATTTCTCC-3′; and Clover: 5′-AGGCCTCTGAGG CCCCTGCAGGCACCATGGTGAGCAAGGGCGAG-3′ and 5′-AGGCCTGACAGGCCG CGGCCGCGCCACCTCCGCTTCCACCTC-3′. pSBtet-Pur was a gift from Eric Kowarz (Addgene plasmid # 60507; http://n2t.net/addgene:60507, accessed on 19 June 2021; RRID:Addgene_60507). Vectors for transient expression of Regnase-1 and for Dox-inducible expression of Regnase-2 were already described [7]. The luciferase constructs (pmirGLO_IL-6-3′UTR, pmirGLO_IL-6-3′UTRΔCE, pmirGLO_ZC3H12A-3′UTR, pmirGLO_TNF-3′UTR and pmirGLO_IER3-3′UTR) were described previously [6,29,38]. The 3′UTRs of IL-1B, IL-12p40 and IL-23A were amplified by PCR using the Q5 Hot Start High-Fidelity 2X Master Mix and cloned into the pmirGLO vector (Promega, Madison, WI, USA) using traditional cloning techniques. The IL-1B 3′UTR was amplified from human cDNA mix with the following primers: 5′-TATGAGCTCAGAGAGCTGTACCCAGAG-3′ and 5′- ATTACTCGAGTCAGAACCATT GAACAGTATG-3′. The IL-12p40 and IL-23A 3′UTRs were amplified from Human Genomic DNA (Clontech, Mountain View, CA, USA) using nested PCR and the following primers: IL-12p40: 5′- GGCCTTCTGGCATGAAATCCCTG-3′, 5′- GACATCCCCTGAGCTCAGGAAGAC-3′ (flanking) and 5′- atagctagcGTTCTGATCC AGGATGAAAATTTGG-3′, 5′- atatctcgagTATGATTACAAAGAAGAGTTTTTATTAG-3′ (nested); IL-23A 5′-TCAGCAGATTCCAAGCCTCAGTCC-3′, 5′-CCATTCTGCTTGCTC AGCCATATAGG-3′ (flanking) and 5′- atagctagcAGGCAGCAGCTCAAGGATGG-3′, 5′- atatctcgagTAGCCACAAAAATAAGACTTTATTG-3′ (nested). The sequences of all used constructs were verified by Sanger sequencing (Genomed, Warszawa, Poland) and the expression of the recombinant proteins was confirmed by Western blotting analysis. All constructs generated in this study are available upon request from the corresponding author.

### 4.9. Luciferase Assays

U251-MG cells were transfected in 24-well plates with 0.8 µg of total DNA used per well. 0.4 µg of an appropriate pmirGLO vector and varying amounts of Regnase-3 (wild type or muteins) or Regnase-1 expression vectors (exact amounts are indicated in figure legends) were used. The amount of DNA per well was equalized using mock plasmid DNA (pcDNA3). 24 h after transfection the cells were lysed and assayed for firefly and Renilla luciferase activity using the Dual-Luciferase Reporter Assay System (Promega, Madison, WI, USA). Renilla luciferase served as internal control.

### 4.10. RNA Isolation and Reverse Transcription

Total RNA isolation was performed according to Chomczynski’s protocol [39]. RNA quantification and purity were assessed using a NanoDrop ND-1000 spectrophotometer (Thermo Fisher Scientific, Wilmington, DE, USA). RNA integrity was analyzed by denaturing, formaldehyde gel electrophoresis. 1.0 µg of RNA was used for reverse-transcription reaction with M-MLV-Reverse transcriptase (Promega) and 500 ng of oligo(dT) primers (or random hexamers for RNA immunoprecipitation procedure) according to the manufacturer’s instructions. For patient derived material the RNA was converted into cDNA using NxGen M-MuLV reverse transcriptase (Lucigen, Middleton, WI, USA) with random and oligo(dT) primers. The cDNA was used in quantitative PCR (RT-qPCR) for the evaluation of the amounts of the mRNAs of interest.

### 4.11. Quantitative PCR (RT-qPCR)

RT-qPCR was performed using RT-HS-PCR-Mix-SYBR-A (A&A Biotechnology). Levels of the mRNAs of interest in each sample were analyzed in duplicates and the expression level was normalized to *POLR2A* or *GAPDH* for human samples and *Eef2* and *cyclophilin A* for mouse samples. Expression levels were analyzed by the ΔΔCt method. Following primers were used—human genes: *Regnase-3* (5′-GTTCACGCCATCCCGGCGAG-3′ and 5′-GGGGCCATGTCTGCCAAGAGG-3′), *Regnase-1* (5′-GGAAGCAGCCGTGTCCCTATG-3′ and 5′-TCCAGGCTGCACTGC TCACTC-3′), *TNF* (5′- CTTCTCGAACCCCGAGTGAC-3′ and 5′- ATGAGGTACA GGCCCTCTGA-3′), *KRT1* (5′-CATCAAGAAGGATGTGGATGGTG-3′ and 5′-GCTTGGT AGAGTGCTGTAAGGAA-3′), *POLR2A* (5′-GCACCACGTCCAATGACAT-3′ and 5′-GTGCGGCTGCTTCCATAA-3′), *GAPDH* (5′-GAGTCAACGGATTTGGTCGTATTG-3′ and 5′- ATGTAGTTGAGGTCAATGAAGGGG-3′); mouse genes: Eef2 (5′-GACATC ACCAAGGGTGTGCAG-3′ and 5′-TTCAGCACACTGGCATAGAGGC-3′), cyclophilin A (5′-AGCATACAGGTCCTGGCATCTTGT-3′ and 5′-CAAAGACCACATGCTTGCCAT CCA-3′) and *Tnf* (5′-AGGCACTCCCCCAAAAGATG-3′ and 5′-CCATTTGGGAACTT CTCATCCC-3′). For detection of mouse *Regnase-3* and *Regnase-1* PrimePCR™ SYBR^®^ Green Assay (Bio-Rad, Des Plaines, IL, USA) primers were used.

### 4.12. Western Blot Analysis

U251-MG cells transfected with appropriate expression vectors were lysed in 1x sample loading buffer (62.5 mM Tris pH8.0, 0.2% (*w*/*v*) SDS, 0.25% (*v*/*v*) β-mercaptoethanol, 10% (*v*/*v*) glycerol; 0.01% (*w*/*v*) bromophenol blue) and the obtained lysates were separated on SDS-PAGE electrophoresis, transferred to PVDF membrane (Millipore) and analyzed by Western blot. Following antibodies were used: anti-FLAG tag (clone M2, Sigma Aldrich, St. Louis, MO, USA), anti-β-actin, anti-mouse-HRP and anti-rabbit HRP (Cell Signaling, Danvers, MA, USA). Luminescence was detected using Clarity Western ECL Substrate (Bio-Rad, Des Plaines, IL, USA) and recorded using the Fusion-Fx documentation system (Vilber Lourmat, Collégien, France).

### 4.13. Immunofluorescence

U251-MG cells were plated on glass coverslips in 12-well culture plates. The following day the cells were transfected with appropriate plasmids (for transient transfection panel) or induced with doxycycline (for stable transfection panel). 24 h post-transfection/induction the cells were fixed in PBS with 4% (*w*/*v*) methanol-free formaldehyde (Thermo Fisher Scientific) for 15 min at room temperature (RT). After fixation the cells were washed 3× with PBS, blocked and permeabilized for 1 h at RT in blocking buffer (5% fetal bovine serum, 0.3% Triton X-100 in PBS). After blocking the cells were incubated overnight at 4 °C in a humidified chamber, in the dark with anti-FLAG tag antibodies (clone M2, Sigma Aldrich, St. Louis, MO, USA) diluted in blocking buffer. The following day cells were washed with PBS and incubated for 90 min at RT in the dark with Alexa Fluor 546-conjugated goat anti-mouse antibody (A-11018, Thermo Fisher Scientific) diluted in blocking buffer. After incubation the cells were washed 3× with PBS, stained with DAPI (Thermo Fisher Scientific) and the samples were mounted onto slides in ProLong Glass Antifade Mountant (Thermo Fisher Scientific). For Clover fluorescent protein fusions after fixation the cells were directly stained with DAPI and mounted onto slides. After overnight curing the samples were imaged using Leica DM6B upright widefield fluorescence microscope (Leica Microsystems, Wetzlar, Germany). All images were acquired using a high numerical aperture 63× oil immersion objective and a 12-bit Leica DMC5400 CMOS camera (Leica Microsystems) with Leica LAS X image acquisition software. The following filter sets (Leica Microsystems) were used: A4 for detection of DAPI, L5 for detection of Clover-fusion proteins, and RHOD ET for Alexa Fluor 546 Dye. After deconvolution from about 30 z-sections with 0.3 μm spacing, images were deconvolved using Huygens Professional Software (Scientific Volume Imaging, Hilversum, Netherlands). Final image adjustments were performed using ImageJ 1.53c (National Institutes of Health, Bethesda, MD, USA) and Adobe Illustrator 2020 (Adobe Systems, Mountain View, CA, USA).

### 4.14. Statistics

Statistical analysis was performed using Graph Pad Prism software (version 5.01, GraphPad Software Inc., San Diego, CA, USA). If not specified differently the asterisks denote statistical significance of the indicated data point versus the control sample. The exact tests performed are indicated in figure descriptions.

## Data Availability

Not applicable.
